# Hydralazine Associated With Reduced Therapeutic Phlebotomy Frequency in a Nationwide Cohort Study: Real-World Effectiveness for Drug Repurposing

**DOI:** 10.3389/fphar.2022.850045

**Published:** 2022-04-01

**Authors:** Wei-Zhi Lin, Chi-Hsiang Chung, Chia-Yang Shaiu, Bing-Heng Yang, Wu-Chien Chien

**Affiliations:** ^1^ Graduate Institute of Life Sciences, National Defense Medical Center, Taipei, Taiwan; ^2^ School of Public Health, National Defense Medical Center, Taipei, Taiwan; ^3^ Department of Medical Research, Tri-Service General Hospital, Taipei, Taiwan; ^4^ Taiwanese Injury Prevention and Safety Promotion Association, Taipei, Taiwan; ^5^ National Defense Medical Center, Graduate Institute of Medical Sciences, Taipei, Taiwan; ^6^ Fidelity Regulation Therapeutics Inc., Taoyuan, Taiwan; ^7^ Division of Clinical Pathology, Department of Pathology, Tri-Service General Hospital, Taipei, Taiwan

**Keywords:** hydralazine, valproate, therapeutic phlebotomy, cohort study, population-based study, national health insurance database

## Abstract

**Background:** Therapeutic phlebotomy, known as scheduled bloodletting, has been the main method for managing erythrocytosis symptoms and thrombocytosis-associated complications in various blood disorders. One of the major indications for phlebotomy is polycythemia vera (PV). The main goal of current treatment strategies for patients who require phlebotomy is to prevent thrombohemorrhagic complications rather than to prolong survival or lessen the risk of myelofibrotic or leukemic progression. Additional cytoreductive therapy is recommended for high-risk PV, for which the common first-line drug is hydroxyurea. However, recent evidence suggests that phlebotomy may not reduce the risk of thrombosis in patients with PV. Further evidence suggests that patients with PV treated with hydroxyurea who require three or more phlebotomy procedures per year have a higher risk of thrombotic complications.

**Methods:** We hypothesized that a drug-repurposing strategy of utilizing antineoplastic drugs for patients who require phlebotomy would result in greater benefits than would phlebotomy. The antihypertensive hydralazine and the anticonvulsant valproate, which have both been reported to have antineoplastic activity that mimics cytoreductive agents, were selected as candidates for the drug-repositioning strategy in a retrospective cohort study. We measured the hazard ratios (HR) and the frequencies of phlebotomy in patients with prescriptions for hydralazine or valproate or the two drugs in combination by using data from Taiwan’s National Health Insurance Research Database from 2000 to 2015 (*n* = 1,936,512).

**Results:** The HRs of undergoing phlebotomy in groups with hydralazine, valproate, and combination hydralazine–valproate prescriptions were reduced to 0.729 (*p* = 0.047), 0.887 (*p* = 0.196), and 0.621 (*p* = 0.022), respectively. The frequency of undergoing phlebotomy decreased from 2.27 to 1.99, 2.01, and 1.86 per person-year (*p* = 0.015), respectively. However, no significant differences were observed for the hydralazine group or the hydralazine–valproate combination group.

**Conclusion:** Whether a repurposed drug can serve as a cytoreductive agent for patients who require phlebotomy depends on its risk–benefit balance. We suggest that hydralazine, instead of the hydralazine–valproate combination, is a reasonable alternative for patients who require regular phlebotomy.

## Introduction

Polycythemia vera (PV) is a neoplastic marrow disorder characterized by the overproduction of red blood cells that affects up to 2.2 persons per 10,000 individuals (Ma et al., 2008; Arber et al., 2016). Currently, few treatment options exist for PV, and patients face the risk of leukemia transformation and myelofibrotic transformation (Cerquozzi et al., 2017; Landtblom et al., 2018). Patients aged under 60 who have no history of thrombosis are classified as a low-risk population. Such patients receive low-dose aspirin as a front-line treatment to prevent thrombotic complications. Therapeutic phlebotomy is performed if these patients’ hematocrit content is higher than 45% (Assi and Baz, 2014; Kim et al., 2021). However, recent evidence suggests that phlebotomy may not reduce the risk of thrombosis in PV (Barbui et al., 2017). Patients aged over 60 years or who have a prior history of thrombosis are classified as a high-risk population. For such patients, the use of hydroxyurea, a chemotherapy drug that inhibits the abnormal proliferation of blood cells (Kim et al., 2021), is recommended. However, some patients cannot tolerate it, and it is also considered to be a risk factor for leukemia transformation or even death (Alvarez-Larran et al., 2012; Cerquozzi et al., 2017). Furthermore, patients with PV treated with hydroxyurea who require three or more phlebotomy procedures per year have a higher risk of thrombotic complications (Alvarez-Larrán et al., 2017). JAK2 and its downstream signal transducer and activator of transcription pathway are known to be abnormally active in PV. This is caused by an increasing JAK2 copy number and a frequently acquired variant, JAK2V617F, which is carried by 95% of those with PV (Campbell et al., 2005; Levine et al., 2005; Scott et al., 2007). Several JAK2 inhibitors, such as fedratinib (Talpaz and Kiladjian, 2021) and ruxolitinib (Mascarenhas and Hoffman, 2012), have been employed in clinical settings but only serve as second-line agents for high-risk patients. Therefore, no ideal treatment options exist for either high-risk or low-risk patients.

Limited treatment options exist for low-risk PV patients despite their elevated risk of thrombosis (∼22%), leukemic, or myelofibrotic transformation [∼18%; (Cerquozzi et al., 2017)]. Phlebotomy is a conservative treatment that simply removes excessive blood cells, and many adverse effects such as the vessel–vagal reflex and vessel failure can develop after long-term phlebotomy. Therefore, the development of medications to slow disease progression and manage hematocrit in low-risk PV patients remains necessary. This study explored whether alternative medications with antineoplastic or cytoreductive potential exist to slow disease progression.

Drug repositioning is an approach that rapidly repurposes developed compounds or marketed drugs to a new indication on the basis of findings from existing data. The approach can be used to rapidly establish a foundation for the safety, dose range, and pharmacokinetic/pharmacodynamic properties of the drug for the indication of interest (Breckenridge and Jacob, 2019; Pushpakom et al., 2019). As an example of drug repositioning, combinations of DNA methyltransferase inhibitors and histone deacetylase inhibitors have been considered as a strategy for epigenetic therapy in cancer (Pathania et al., 2016). Though the use of therapeutic phlebotomy is commonly employed in PV patients, PV patients were just small portion of participants enrolled in our study. The present study selected hydralazine and valproate as candidates for a drug-repositioning strategy to treat patients who require phlebotomy in this cohort reflecting the whole Taiwan population. Hydralazine was originally used for hypertension management, acting as a known DNA methyltransferase inhibitor (Deng et al., 2003). Valproate is an antipsychotic agent widely used for epilepsy and affective psychosis and was reported to be a histone deacetylase inhibitor (Phiel et al., 2001). The combination of hydralazine and valproate has been studied for various hematological malignancies such as mycosis fungoides (Duenas-Gonzalez et al., 2010), myelodysplastic syndrome (Candelaria et al., 2011; Candelaria et al., 2017), cutaneous T-cell lymphoma (Espinoza-Zamora et al., 2017; Schcolnik-Cabrera et al., 2018), and myeloid leukemia (Cervera et al., 2012; Lubbert et al., 2020). Previous study also demonstrated that hydralazine may have potential of reducing risk of developing to several subgroups of hematologic neoplasms (Yang et al., 2022).

Currently, no pharmacoepidemiological study utilizing hydralazine and valproate as candidates of drug repurposing for patients who require phlebotomy has been reported. We attempted to validate the potential of hydralazine and valproate in a nationwide cohort by using data from the Taiwan National Health Insurance Research Database from 2000 to 2015 (NHIRD 2000–2015). We calculated the differences in the hazard ratios (HRs) and frequencies of therapeutic phlebotomy for patients with and without hydralazine, valproate, and combination hydralazine–valproate prescriptions.

## Materials and Methods

### Ethics Approval and Consent to Participate

The personal identification data from NHIRD 2000-2015 were encrypted to protect privacy. The protocol of this study was reviewed and approved by the Institutional Review Board of the Tri-Service General Hospital (No.: B-109-38).

### Data Source

Data were retrospectively collected from the NHIRD 2000–2015. The Taiwan National Health Insurance program was launched in 1995 and most of the Taiwan population are enrolled (Lin et al., 2018). The NHIRD is a representative cohort that contains detailed registry and claims data, including data from outpatient departments and inpatient hospital care settings from the National Health Insurance Program. The NHIRD collects basic demographic information (such as sex, birthday, and area of residence), insurance premium, prescriptions, operations, examinations, medical visits, and disease diagnoses according to the *International Classification of Diseases, 9th Revision, Clinical Modification* (*ICD-9-CM*) codes which were all made by board-certified clinicians. The National Health Insurance Administration regularly, retrospectively and randomly reviews the medical records in the NHIRD to verify the accuracy of the diagnoses and that appropriate management was provided. All personally identifying information in the NHIRD was obscured to protect patient privacy. Previous study reported a high quality (94% accuracy) of principal diagnosis (Cheng et al., 2011) in comparison with medical records in one medical center, indicating the accurate database in NHIRD.

### Sampled Patients and Outcome Measures

Patients who received the candidate drugs (hydralazine or valproate) were included. Patients who were younger than 20 years, received hydralazine or valproate continuously for less than 180 days, lacked a listed date at which they started receiving the candidate drugs, met the *ICD-9-CM* diagnostic criteria for malignant neoplasms of lymphatic and hematopoietic tissue, or received therapeutic phlebotomy before tracking were excluded. A control group of patients without prescriptions of hydralazine or valproate were matched to patients in the experimental group in a 4:1 ratio in study groups according to age, sex, and index year.

To study the dose-dependent effect of candidate drugs on the occurrence of therapeutic phlebotomy, a stratified analysis was conducted for five dose levels, namely 0%–19%, 20%–39%, 40%–59%, 60%–79%, and 80–100% of the defined daily dose (DDD), which is 300 mg per day for hydralazine and 2000 mg per day for valproate according to maximum daily consumption. The included patients were followed up until the end of the study period (end of 2015). The duration of follow-up represents the interval between the date of inclusion and the date the patient underwent their first therapeutic phlebotomy (*ICD-9-CM*: 94004C). Subsequently, the frequency that the patients received therapeutic phlebotomy was monitored until the end of the study period.

### Covariates

The covariates were sex, age groups (20–29, 30–39, 40–49, 50–59, and over 60 years), area of residence (north, central, south, and east Taiwan), level of hospital (medical center, regional hospital, local hospital), and urbanization level of the town of residence (levels 1–4). The urbanization level of the area of residence was defined according to population and several indicators of development. Level 1 was defined as a region with a population of more than 1,250,000 and with a specific designation as a political, economic, cultural, and metropolitan center; level 2 was defined as a region with a population of 500,000 to 1,249,999 and that plays a key role in politics, economy, and culture; levels 3 and 4 were defined as regions with populations of 150,000 to 499,999 and under 149,999, respectively.

The comorbidities were hypertension (*ICD-9-CM*: 401–405), gestational hypertension (*ICD-9-CM*: 642.0–642.3, 642.7, 642.9), idiopathic pulmonary artery hypertension (*ICD-9-CM:* 416.0), congestive heart failure (*ICD-9-CM*: 428), affective psychosis (*ICD-9-CM*: 296), epilepsy (*ICD-9-CM*: 345), migraine (*ICD-9-CM*: 346), pulmonary embolism (PE, *ICD-9-CM*: 415.1), gastric ulcer (*ICD-9-CM*: 531), peptic ulcer disease (*ICD-9-CM*: 533), gastrojejunal ulcer (*ICD-9-CM*: 534), gastrointestinal hemorrhage (GI hemorrhage, *ICD-9-CM*: 578), Budd–Chiari syndrome (*ICD-9-CM*: 453.0), cerebral thrombosis (*ICD-9-CM*: 434.0), ischemic heart disease (*ICD-9-CM*: 411), vascular insufficiency of intestine (*ICD-9-CM*: 557), and Charlson comorbidity index with the aforementioned diseases removed (CCI_R; Supplementary Table S1).

### Statistical Analysis

The results are presented as HRs with a 95% confidence interval, adjusted for the aforementioned covariates by using multivariate Cox regression analysis. The differences between the four groups (control, hydralazine, valproate, and hydralazine–valproate in combination) were calculated using the Kaplan-Meier method with the log-rank test or the Scheffe post hoc test. The chi-square test was used to compare categorical variables by treatment types when the categorical outcomes were larger than five, and Fisher’s exact test was used when the categorical outcomes were smaller than five. A two-tailed *p* value of < 0.05 was considered significant. All statistical analyses were performed using SPSS (version 22.0, IBM Corp., Armonk, NY, United States).

## Results

### Patients Enrolled

**FIGURE 1:**
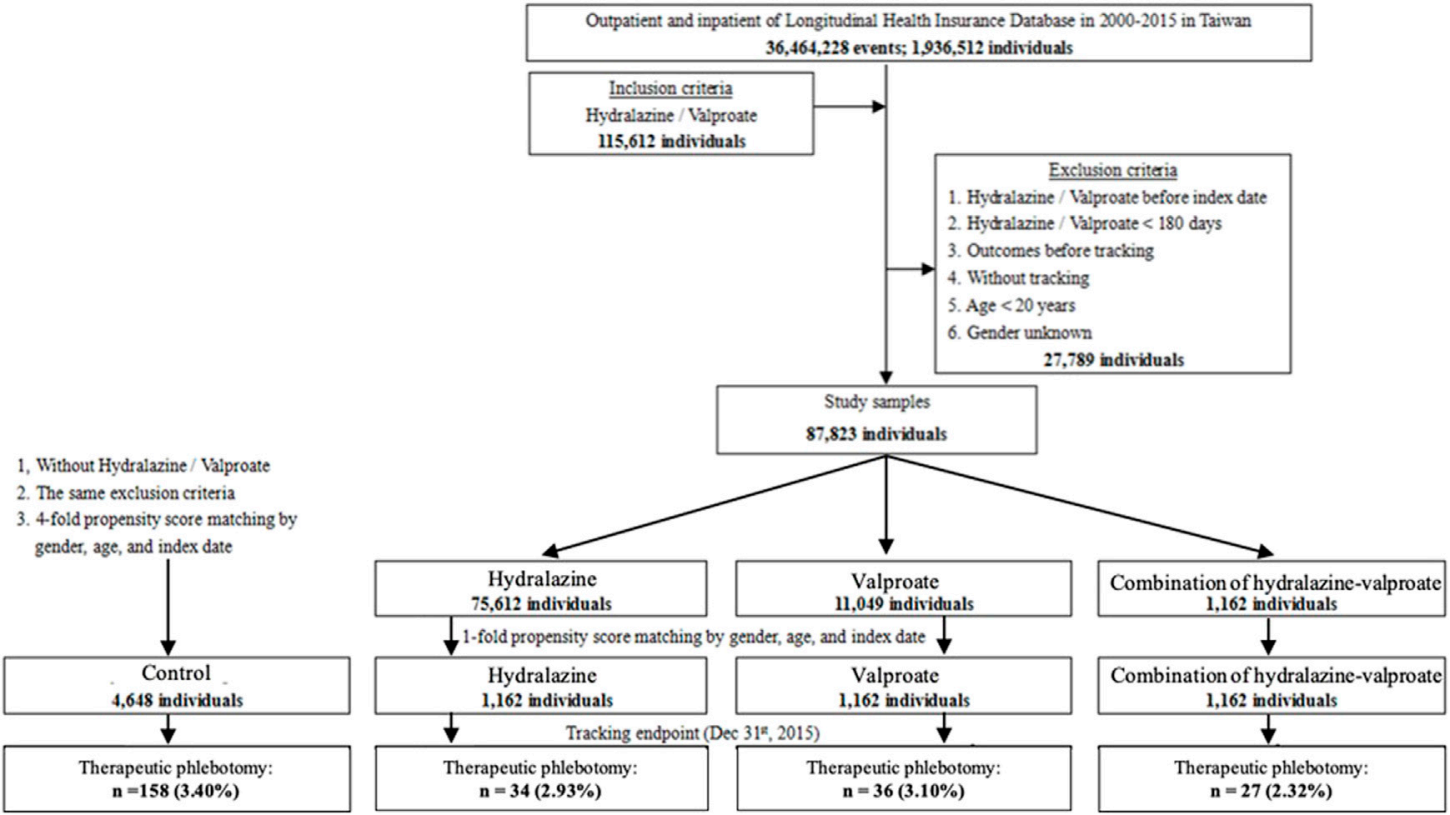
Flowchart of patient enrollment.

A total of 115,612 patients were initially included, of which 27,789 were excluded according to the aforementioned exclusion criteria. Of the remaining 87,823 patients, 75,612 had received a hydralazine prescription, 11,049 had received a valproate prescription, and the remainder (1,162 patients) had received a prescription for both hydralazine and valproate. Two subgroups of 1,162 patients each were randomly created from the hydralazine group and the valproate group. A total of 4,648 enrollees who did not take hydralazine or valproate were selected as controls (Figure 1).

The sex ratio (male/female) of patients was 1.15. More than half of the patients were aged >60 years. The percentage of patients with a history of hypertension in the hydralazine group was significantly higher than that in the control and the valproate groups because hypertension is an indication for hydralazine (*p* < 0.001). Similarly, the percentage of patients in the valproate group with a history of affective psychosis, epilepsy, or migraine was significantly higher than that in the control and hydralazine groups (*p* < 0.001). The prevalence of gastric and gastrojejunal ulcer was lower in the valproate and combination groups (*p* < 0.001 for gastric ulcer; *p* = 0.010 for gastrojejunal ulcer). The prevalence of cerebral thrombosis was significantly higher in the combination group (*p* < 0.001). Patients in the hydralazine and combination groups had a higher CCI_R score than those in other groups (*p* < 0.001). In addition, more than 70% of the patients were residents in cities with a high urbanization level (level 1–2). The patients were most likely to be treated in a local hospital, especially those in the hydralazine group (Supplementary Table S2).

### Factors Correlated With Therapeutic Phlebotomy

In our cohort, patients were more likely to receive therapeutic phlebotomy at higher-level hospitals, in cities with higher levels of urbanization, and between winter and spring. Male patients had a lower risk of meeting the criteria for receiving therapeutic phlebotomy (adjusted HR = 0.766; *p* = 0.038). Patients with hypertension, affective psychosis, gastrojejunal ulcer, GI hemorrhage, ischemic heart disease, and other diseases or conditions included in the Charlson comorbidity index were high-risk populations for receiving therapeutic phlebotomy (*p* < 0.05). By contrast, patients with epilepsy had a lower risk of receiving therapeutic phlebotomy (*p* = 0.042; Table 1).

**TABLE 1:** Factors affecting risk of requiring therapeutic phlebotomy determined using Cox regression.

Variables	Adjusted HR	95% CI		*p*
Group				
Control	Reference			
Hydralazine	0.729	0.572	0.991	0.047
Valproate	0.887	0.548	1.131	0.196
Combination of hydralazine-valproate	0.621	0.413	0.934	0.022
Gender				
Male	0.766	0.595	0.985	0.038
Female	Reference			
Age group (yrs)				
20-29	Reference			
30-39	1.562	0.173	4.161	0.992
40-49	2.451	0.134	5.453	0.902
50-59	1.284	0.045	4.27	0.917
≧60	0.986	0.127	5.567	0.903
Season				
Spring	Reference			
Summer	0.633	0.442	0.907	0.013
Autumn	0.496	0.344	0.715	<0.001
Winter	0.907	0.657	1.253	0.554
Urbanization level				
1 (The highest)	1.54	1.009	2.349	0.045
2	1.427	0.968	2.102	0.072
3	1.147	0.627	2.096	0.657
4 (The lowest)	Reference			
Levels of hospitals				
Hospital center	1.246	0.856	1.814	0.250
Regional hospital	1.126	0.806	1.574	0.486
Local hospital	Reference			
Hypertension	1.452	1.326	1.626	<0.001
Gestational Hypertension	0	—	—	0.999
IPAH	1.128	0.114	6.035	0.852
Congestive heart failure	0.961	0.61	1.512	0.862
Affective psychosis	1.199	1.012	1.65	0.017
Epilepsy	0.807	0.326	1.994	0.042
Migraine	0	—	—	0.870
PE	0.863	0.117	6.367	0.885
Gastric ulcer	1.557	0.865	2.804	0.140
Peptic ulcer disease	0.989	0.312	3.132	0.985
Gastrojejunal ulcer	2.11	1.386	5.511	<0.001
GI hemorrhage	2.039	1.24	3.354	0.005
Budd–Chiari syndrome	0.826	0.115	5.935	0.850
Cerebral thrombosis	1.303	0.124	2.171	0.249
Ischemic heart disease	2.532	1.117	5.744	0.026
Vascular insufficiency of intestine	1.69	0.413	6.909	0.465
CCI_R	1.786	1.615	1.976	<0.001

HR, hazard ratio; CI, confidence interval; Adjusted HR, adjusted variables listed in the table, Location had multicollinearity with urbanization level, IPAH, idiopathic pulmonary artery hypertension.

### Reduced Cumulative Risk and Decreased Frequency of Therapeutic Phlebotomy

**FIGURE 2:**
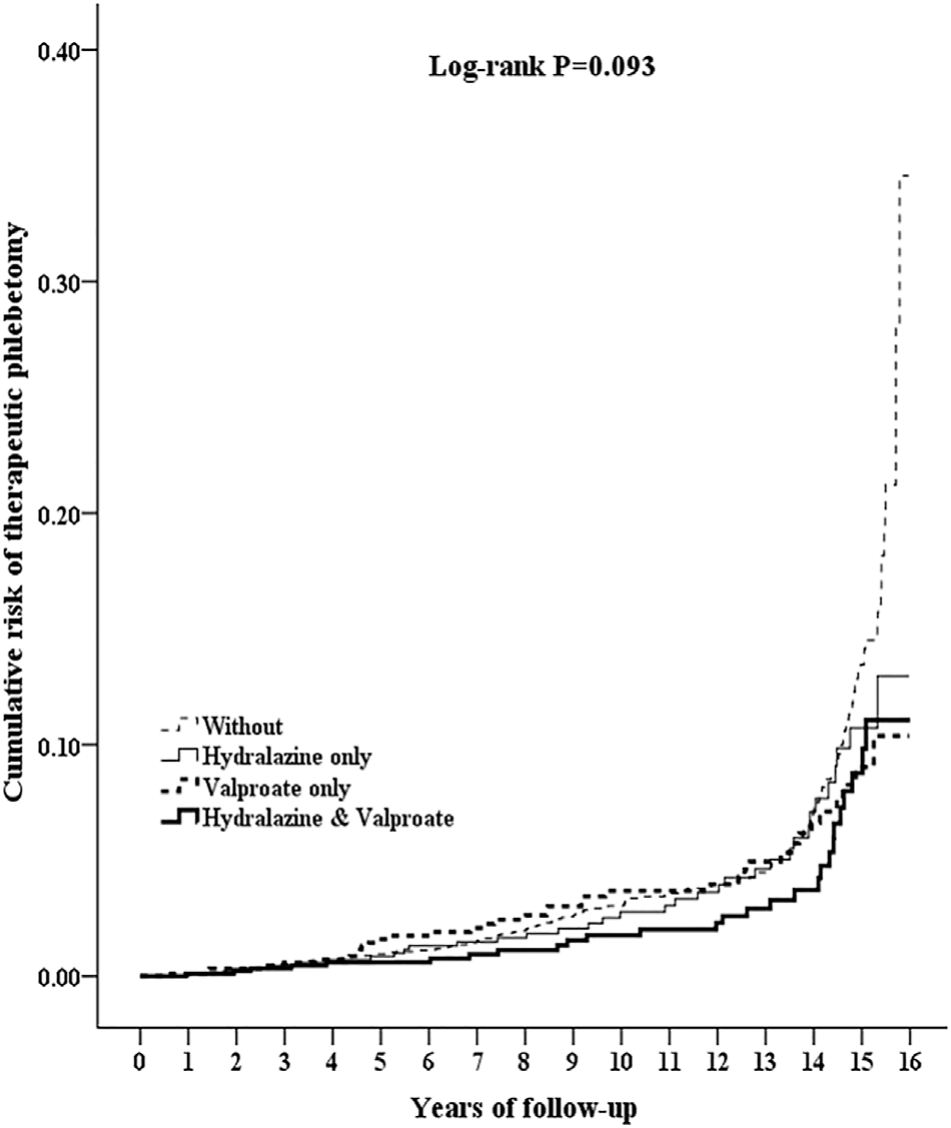
Kaplan–Meier curves for cumulative risk of requiring therapeutic phlebotomy in patients aged ≥20 from different cohorts drawn using the log-rank test. Log-rank test: Control vs. hydralazine, *p* = 0.058; control vs. valproate, *p* = 0.185; control vs. combination hydralazine–valproate, *p* = 0.018; hydralazine vs. valproate, *p* = 0.971; hydralazine vs. combination hydralazine–valproate, *p* = 0.258; valproate vs. combination hydralazine–valproate, *p* = 0.318.

Patients with a combination hydralazine–valproate prescription had a lower cumulative occurrence of therapeutic phlebotomy than did the control, but occurrence in the hydralazine and valproate groups was not significantly lower than that in the control (*p* = 0.024 for the combination group vs. the control, 0.058 for the hydralazine group vs. the control, 0.185 for the valproate group vs. the control). No significant difference in occurrence was observed among the hydralazine, valproate, or combination groups (*p* = 0.258–0.971; Figure 2).

The frequency of therapeutic phlebotomy in the hydralazine, valproate, and combination groups was significantly lower than that in the control group (*p* = 0.015), whereas no significant differences were observed among the hydralazine, valproate, and combination groups (Table 2).

**TABLE 2:** Frequency of therapeutic phlebotomy in different groups.

Group	1. Control		2. Hydralazine		3. Valproate		4. Combination of Hydralazine-Valproate		*P*	
Outcomes	*n*	Mean ± SD, per PY	*n*	Mean ± SD, per PY	*n*	Mean ± SD, per PY	*n*	Mean ± SD, per PY		Scheffe post hoc
Phlebotomy	158	2.27 ± 3.45	34	1.99 ± 2.47	36	2.01 ± 3.26	27	1.86 ± 2.98	0.015	1 > 3 = 2 = 4

*P:* One-way ANOVA with Scheffe post hoc.

### Decreasing HR of Therapeutic Phlebotomy Stratified by Prescription Dose

In the hydralazine group, the HR of undergoing therapeutic phlebotomy was significantly lower even under a low dose (<20% DDD, *p* = 0.040). The HR in the overall hydralazine group was close to the boundary of statistical significance (*p* = 0.047). In the valproate group, only the subgroup with a high dose (>60% DDD) had a significantly lower HR (60–79% DDD; *p* = 0.047; ≥80%; *p* = 0.012). A similar pattern was observed in the combination group; the HR was significantly lower in subgroups with a dose higher than 60% (*p* < 0.001). The dose-dependent effect was strong in the valproate and combination groups but was absent in the hydralazine group (Table 3).

**TABLE 3:** HR of therapeutic phlebotomy stratified by prescription dose.

Group, Dose (DDD)	Population	Events	PYs	Rate (per 10^5^ PYs)	Adjusted HR	95% CI		*P*
Control	4,648	158	48,055.71	328.79	Reference			
Hydralazine	1,162	34	12,653.22	268.71	0.729	0.572	0.991	0.047
<20%	321	8	3,495.43	228.87	0.621	0.487	0.844	0.040
20-39%	204	7	2,221.39	315.12	0.855	0.671	1.163	0.055
40-59%	334	9	3,636.98	247.46	0.674	0.523	0.912	0.033
60-79%	155	6	1,687.82	355.49	0.964	0.754	1.313	0.062
≧80%	148	4	1,611.60	248.20	0.673	0.521	0.915	0.036
Valproate	1,162	36	12,707.30	283.30	0.887	0.548	1.131	0.196
<20%	287	8	3,138.55	254.89	0.798	0.490	1.026	0.178
20-39%	219	9	2,394.92	375.80	1.076	0.723	1.512	0.226
40-59%	301	10	3,291.65	303.80	0.942	0.587	1.218	0.203
60-79%	189	5	2,066.85	241.91	0.757	0.465	0.996	0.047
≧80%	166	4	1,815.33	220.35	0.691	0.423	0.880	0.012
Combination of hydralazine-valproate	1,162	27	12,754.98	211.68	0.621	0.413	0.934	0.022
<20%	105	4	1,284.90	311.31	0.842	0.682	1.112	0.142
20-39%	119	4	1,229.75	325.27	0.942	0.735	1.267	0.206
40-59%	197	6	2,038.15	294.38	0.758	0.595	1.086	0.078
60-79%	158	3	1,683.45	178.21	0.582	0.297	0.864	<0.001
≧80%	583	10	6,518.73	153.40	0.429	0.198	0.726	<0.001

PYs, Person-years.

Adjusted HR, adjusted hazard ratio, adjusted by the variates listed in Table 1.

CI, confidence interval.

DDD for hydralazine = 300 mg per day, for valproate = 2000 mg per day.

### Limitations

This retrospective cohort study was based on the NHIRD and employed *ICD-9-CM* codes; thus, some of the data may be inaccurate. For example, the dose level of treatment was estimated by dividing the cumulative doses of individual medications by the prescription duration. Several indexes such as the volume of phlebotomy, hematocrit content, and the genotype of the oncogene, such as JAK2V617F, were not recorded. Furthermore, body mass index, real income, and lifestyle factors, including smoking/drinking frequency and dietary factors, were not recorded in the NHIRD. The patients whose data are contained in the NHIRD were assumed to be ethnic Taiwanese, with considerable similarity to Southern Han Chinese; a very small portion of the patients may not be ethnic Taiwanese, such as immigrants or foreign residents.

## Discussion

For unknown reasons, male patients exhibited a lower occurrence of therapeutic phlebotomy, and therapeutic phlebotomy procedures were most commonly performed in the winter and spring. Whether sex or seasonal factors are correlated with therapeutic phlebotomy merits further study. Some diseases were significantly correlated with a higher rate of therapeutic phlebotomy, especially gastrojejunal ulcer (*p* < 0.001), GI hemorrhage (*p* = 0.005), and hypertension (*p* < 0.001). The contracted plasma volume caused by excess blood cells in PV patients may lead to hypertension (Zeis et al., 1979), and PV may increase the risk of thrombosis, which is typically managed with aspirin. The long-term use of aspirin may induce gastrojejunal ulcer and GI hemorrhage (Cryer and Mahaffey, 2014). Furthermore, gastrojejunal ulcer and GI hemorrhage are common symptoms in patients with PV because of the abnormally high release of histamine by mast cells or increased susceptibility to *H. pylori* infection (Gilbert et al., 1966; Torgano et al., 2002). In addition, psychosis is associated with therapeutic phlebotomy (adjusted HR = 1.199; *p* = 0.017). A model has been proposed to explain psychiatric events resulting from blood hyperviscosity, including slowed blood flow with hypoxia and small, multiple thromboses in the central nervous system (Coelho et al., 2022). Ischemic heart disease was also observed to be associated with frequent therapeutic phlebotomy (adjusted HR = 2.532; *p* = 0.026), which may be caused by the correlation between cyanotic congenital heart disease and secondary polycythemia (Assi and Baz, 2014). Therefore, the aforementioned diseases should be considered comorbidities but not contributing factors.

Patients with epilepsy, which is the original indication of valproate, had a low risk of requiring therapeutic phlebotomy (adjusted HR = 0.807; *p* = 0.042), but the mechanism remains unknown; whether the reduced need for therapeutic phlebotomy in the valproate group was caused by epilepsy or valproate requires further study (Table 1).

No dose-dependent effects were observed in the hydralazine group. By contrast, a dose-dependent pattern was observed in the valproate group, but the HRs were significantly lower only for doses up to 60% of DDD (1,200 mg per day). A similar pattern was observed in the combination group (Table 3). This may indicate that the mechanisms of action of hydralazine and valproate are independent of each other. Furthermore, hydralazine was reported to induce lupus syndromes (incidence >5% when doses were up to 100 mg per day) in a cohort study (*n* = 281), whereas no lupus event was observed in a group with doses of 50 mg per day (Cameron and Ramsay, 1984). A lower dose of hydralazine (<20% of DDD, <60 mg per day) might be less likely to cause lupus syndromes and be efficacious in reducing the need for therapeutic phlebotomy (adjusted HR = 0.621; *p* = 0.040; Table 3). We suggest that further dose-finding studies use initial doses of less than 60 mg per day.

In addition to the reduction in phlebotomy frequency, inhibitory efficacy of cell survival rate was also disclosed on leukocytes of chronic myeloproliferative patients after treatment of hydralazine and valproate in previous work (Yang et al., 2022). There are two phlebotomy volume, 250 ml or 500 ml once a time of phlebotomization, applied for patients by Taiwan physician’s order. The therapeutic code of 94004C represents performing once phlebotomization in NHIRD. In spite of the difference between 250 and 500 ml per phlebotomization, our study disclosed that the frequency of undergoing phlebotomy significantly decreased from 2.27 to 1.99 per year in patients with regular prescription of hydralazine. Although the overall results indicate that combination hydralazine–valproate may act as an efficient cytoreductive agent and may ensure greater cytoreductive potential for patients who require therapeutic phlebotomy than hydralazine alone, the combination of the two may not be ideal as an additional treatment for patients who require phlebotomy. In this study, a low proportion of the patients met the criteria to take both hydralazine and valproate (Figure 1), and the standard medication for patients with PV, aspirin, is known to increase plasma concentrations of valproate and hamper its metabolism (Sandson et al., 2006).

Considering the risk–benefit balance of drug repurposing for clinical decision-making, we suggest that hydralazine, instead of combination valproate–hydralazine, could be feasible for patients who require regular phlebotomy.

## Data Availability

The datasets used in this study are available from the Taiwan NHIRD. Because of legal restrictions imposed by the government of Taiwan in relation to the Personal Information Protection Act, data cannot be made publicly available. Requests for data can be sent as a formal proposal to the NHIRD (http://nhird.nhri.org.tw).

## Abbreviations

CCI_R, Charlson comorbidity index without the diseases listed in Section 2.4; DDD, defined daily dose; GI hemorrhage, gastrointestinal hemorrhage; HR, hazard ratio; *ICD-9-CM*, *international classification of diseases, 9th revision, clinical modification*; JAK2, Janus kinase 2; NHIRD, national health insurance research database; PV, polycythemia vera.
