# Supplementary material for: Hydralazine Associated With Reduced Therapeutic Phlebotomy Frequency in a Nationwide Cohort Study: Real-World Effectiveness for Drug Repurposing
Source: Front Pharmacol. 2022 Apr 1;13:850045. doi: 10.3389/fphar.2022.850045 (PMC9011102; doi:10.3389/fphar.2022.850045)
Supplement: Supplementary file 1 [file DataSheet1.docx]

Supplementary Material

### Table S1. Characteristics of study in the baseline

| **Group** | **Overall** | | **1. Without** | | **2. Hydralazine only** | | **3. Valproate only** | | **4. Hydralazine & Valproate** | | ***P*** |
| --- | --- | --- | --- | --- | --- | --- | --- | --- | --- | --- | --- |
| **Variables** | **n** | **%** | **n** | **%** | **n** | **%** | **n** | **%** | **n** | **%** |  |
| **Total** | 8,134 |  | 4,648 |  | 1,162 |  | 1,162 |  | 1,162 |  |  |
| **Gender** | | | | | | | | | | | .999 |
| Male | 4,347 | 53.44 | 2,484 | 53.44 | 621 | 53.44 | 621 | 53.44 | 621 | 53.44 |  |
| Female | 3,787 | 46.56 | 2,164 | 46.56 | 541 | 46.56 | 541 | 46.56 | 541 | 46.56 |  |
| **Age (years)** | 61.14 ± 13.35 | | 61.08 ± 13.45 | | 61.84 ± 13.38 | | 60.56 ± 13.17 | | 61.23 ± 13.13 | | .136 |
| **Age group (yrs)** | | | | | | | | | | | .999 |
| 20-29 | 63 | 0.77 | 36 | 0.77 | 9 | 0.77 | 9 | 0.77 | 9 | 0.77 |  |
| 30-39 | 462 | 5.68 | 264 | 5.68 | 66 | 5.68 | 66 | 5.68 | 66 | 5.68 |  |
| 40-49 | 1,379 | 16.95 | 788 | 16.95 | 197 | 16.95 | 197 | 16.95 | 197 | 16.95 |  |
| 50-59 | 1,512 | 18.59 | 864 | 18.59 | 216 | 18.59 | 216 | 18.59 | 216 | 18.59 |  |
| ≧60 | 4,718 | 58.00 | 2,696 | 58.00 | 674 | 58.00 | 674 | 58.00 | 674 | 58.00 |  |
| **HTN** | | | | | | | | | | | <.001 |
| Without | 6,821 | 83.86 | 4,150 | 89.29 | 777 | 66.87 | 1,038 | 89.33 | 856 | 73.67 |  |
| With | 1,313 | 16.14 | 498 | 10.71 | 385 | 33.13 | 124 | 10.67 | 306 | 26.33 |  |
| **Gestational HTN** | | | | | | | | | | | <.001 |
| Without | 8,123 | 99.86 | 4,646 | 99.96 | 1,154 | 99.31 | 1,162 | 100.00 | 1,161 | 99.91 |  |
| With | 11 | 0.14 | 2 | 0.04 | 8 | 0.69 | 0 | 0.00 | 1 | 0.09 |  |
| **IPAH** | | | | | | | | | | | .025 |
| Without | 8,107 | 99.67 | 4,637 | 99.76 | 1,153 | 99.23 | 1,160 | 99.83 | 1,157 | 99.57 |  |
| With | 27 | 0.33 | 11 | 0.24 | 9 | 0.77 | 2 | 0.17 | 5 | 0.43 |  |
| **CHF** | | | | | | | | | | | .001 |
| Without | 7,942 | 97.64 | 4,539 | 97.65 | 1,118 | 96.21 | 1,146 | 98.62 | 1,139 | 98.02 |  |
| With | 192 | 2.36 | 109 | 2.35 | 44 | 3.79 | 16 | 1.38 | 23 | 1.98 |  |
| **Affective psychosis** | | | | | | | | | | | <.001 |
| Without | 7,458 | 91.69 | 4,503 | 96.88 | 1,140 | 98.11 | 864 | 74.35 | 951 | 81.84 |  |
| With | 676 | 8.31 | 145 | 3.12 | 22 | 1.89 | 298 | 25.65 | 211 | 18.16 |  |
| **Epilepsy** | | | | | | | | | | | <.001 |
| Without | 7,759 | 95.39 | 4,645 | 99.94 | 1,158 | 99.66 | 1,041 | 89.59 | 915 | 78.74 |  |
| With | 375 | 4.61 | 3 | 0.06 | 4 | 0.34 | 121 | 10.41 | 247 | 21.26 |  |
| **Migraine** | | | | | | | | | | | <.001 |
| Without | 7,915 | 97.31 | 4,647 | 99.98 | 1,153 | 99.23 | 1,064 | 91.57 | 1,051 | 90.45 |  |
| With | 219 | 2.69 | 1 | 0.02 | 9 | 0.77 | 98 | 8.43 | 111 | 9.55 |  |
| **PE** | | | | | | | | | | | .929 |
| Without | 8,123 | 99.86 | 4,642 | 99.87 | 1,160 | 99.83 | 1,161 | 99.91 | 1,160 | 99.83 |  |
| With | 11 | 0.14 | 6 | 0.13 | 2 | 0.17 | 1 | 0.09 | 2 | 0.17 |  |
| **Gastric ulcer** | | | | | | | | | | | <.001 |
| Without | 7,862 | 96.66 | 4,470 | 96.17 | 1,116 | 96.04 | 1,132 | 97.42 | 1,144 | 98.45 |  |
| With | 272 | 3.34 | 178 | 3.83 | 46 | 3.96 | 30 | 2.58 | 18 | 1.55 |  |
| **PUD** | | | | | | | | | | | .318 |
| Without | 8,032 | 98.75 | 4,590 | 98.75 | 1,142 | 98.28 | 1,152 | 99.14 | 1,148 | 98.80 |  |
| With | 102 | 1.25 | 58 | 1.25 | 20 | 1.72 | 10 | 0.86 | 14 | 1.20 |  |
| **Gastrojejunal ulcer** | | | | | | | | | | | .010 |
| Without | 8,127 | 99.91 | 4,645 | 99.94 | 1,158 | 99.66 | 1,162 | 100.00 | 1,162 | 100.00 |  |
| With | 7 | 0.09 | 3 | 0.06 | 4 | 0.34 | 0 | 0.00 | 0 | 0.00 |  |
| **GI hemorrhage** | | | | | | | | | | | .081 |
| Without | 8,014 | 98.52 | 4,566 | 98.24 | 1,150 | 98.97 | 1,147 | 98.71 | 1,151 | 99.05 |  |
| With | 120 | 1.48 | 82 | 1.76 | 12 | 1.03 | 15 | 1.29 | 11 | 0.95 |  |
| **Budd-Chiari syndrome** | | | | | | | | | | | .500 |
| Without | 8,123 | 99.86 | 4,640 | 99.83 | 1,162 | 100.00 | 1,161 | 99.91 | 1,160 | 99.83 |  |
| With | 11 | 0.14 | 8 | 0.17 | 0 | 0.00 | 1 | 0.09 | 2 | 0.17 |  |
| **Cerebral thrombosis** | | | | | | | | | | | <.001 |
| Without | 7,746 | 95.23 | 4,566 | 98.24 | 1,069 | 92.00 | 1,088 | 93.63 | 1,023 | 88.04 |  |
| With | 388 | 4.77 | 82 | 1.76 | 93 | 8.00 | 74 | 6.37 | 139 | 11.96 |  |
| **IHD** | | | | | | | | | | | .011 |
| Without | 8,066 | 99.16 | 4,607 | 99.12 | 1,145 | 98.54 | 1,155 | 99.40 | 1,159 | 99.74 |  |
| With | 68 | 0.84 | 41 | 0.88 | 17 | 1.46 | 7 | 0.60 | 3 | 0.26 |  |
| **Vascular insufficiency of intestine** | | | | | | | | | | | .989 |
| Without | 8,128 | 99.93 | 4,645 | 99.94 | 1,161 | 99.91 | 1,161 | 99.91 | 1,161 | 99.91 |  |
| With | 6 | 0.07 | 3 | 0.06 | 1 | 0.09 | 1 | 0.09 | 1 | 0.09 |  |
| **CCI_R** | 0.53 ± 0.91 | | 0.39 ± 0.79 | | 0.85 ± 1.11 | | 0.54 ± 0.92 | | 0.76 ± 0.97 | | <.001 |
| **Urbanization level** | | | | | | | | | | | .009 |
| 1 (The highest) | 2,676 | 32.90 | 1,553 | 33.41 | 375 | 32.27 | 362 | 31.15 | 386 | 33.22 |  |
| 2 | 3,382 | 41.58 | 1,924 | 41.39 | 460 | 39.59 | 513 | 44.15 | 485 | 41.74 |  |
| 3 | 595 | 7.31 | 336 | 7.23 | 117 | 10.07 | 70 | 6.02 | 72 | 6.20 |  |
| 4 (The lowest) | 1,481 | 18.21 | 835 | 17.96 | 210 | 18.07 | 217 | 18.67 | 219 | 18.85 |  |
| **Levels of hospitals** | | | | | | | | | | | <.001 |
| Medical center | 2,508 | 30.83 | 1,495 | 32.16 | 326 | 28.06 | 331 | 28.49 | 356 | 30.64 |  |
| Regional hospital | 2,544 | 31.28 | 1,413 | 30.40 | 358 | 30.81 | 424 | 36.49 | 349 | 30.03 |  |
| Local hospital | 3,082 | 37.89 | 1,740 | 37.44 | 478 | 41.14 | 407 | 35.03 | 457 | 39.33 |  |
| ***P:* Chi-square / Fisher exact test on category variables and One-way ANOVA with Scheffe post hoc on continue variables** | | | | | | | | | | | |

### Table S2. Abbreviation, ICD-9-CM, NHI code and Definition

|  | **Abbreviation** | **ICD-9-CM / NHI code / Definition** |
| --- | --- | --- |
| **Study population:** |  |  |
| Hydralazine |  | Continuous use ≧180 days |
| Valproate |  | Continuous use ≧180 days |
| **Outcomes** |  |  |
| Therapeutic phlebotomy |  | 94004C |
| **Comorbidities** |  |  |
| Hypertension |  | 401-405 |
| Gestational hypertension | Gestational HTN | 642.0-642.3, 642.7, 642.9 |
| Idiopathic pulmonary artery hypertension | IPAH | 416.0 |
| Congestive heart failure |  | 428 |
| Affective psychosis |  | 296 |
| Epilepsy |  | 345 |
| Migraine |  | 346 |
| Pulmonary embolism | PE | 415.1 |
| Gastric ulcer |  | 531 |
| Peptic ulcer disease |  | 533 |
| Gastrojejunal ulcer |  | 534 |
| Gastrointestinal hemorrhage | GI hemorrhage | 578 |
| Budd-Chiari syndrome |  | 453.0 |
| Cerbral thrombosis |  | 434.0 |
| Ischemic heart disease |  | 411 |
| Vascular insufficiency of intestine |  | 557 |
